# Simvastatin Induces Apoptosis And Suppresses Hepatocellular Carcinoma Induced In Rats

**DOI:** 10.1007/s12010-022-04203-0

**Published:** 2022-11-11

**Authors:** Yomna A. Elleithi, Amal M. El-Gayar, Mohamed N. Amin

**Affiliations:** 1 Biochemistry Department, Faculty of Pharmacy, Mansoura University, Mansoura, 3551 Egypt; 2 Biochemistry Department, Faculty of Pharmacy, King Salman International University, Ras Sedr, South Sinai Egypt

**Keywords:** HCC, Simvastatin, Liver, Cancer, In vivo, AFP

## Abstract

Hepatocellular carcinoma (HCC) is a frequent primary aggressive cancer, a crucial cause of cancer-related mortality globally. Simvastatin is a well-known safe cholesterol-lowering medication that has been recently shown to suppress cancer progression. Apoptosis is a well-organized and controlled cellular process that happens both physiologically and pathologically leading to executing cell death. Apoptosis is frequently downregulated in cancer cells. In the present study, we aimed to test the effect of simvastatin on HCC progression. HCC was induced in experimental rats by means of diethylnitrose amine (DEN) and thioacetamide (TAA) injections. Gross examination and liver index along with biochemical analysis of hepatic function were evaluated. Serum alpha-feto protein (AFP) concentration was measured by ELISA. Histopathological examination was used for assessing necroinflammatory scores and fibrosis degree. Apoptosis was assessed using immunohistochemistry (IHC) and quantitative PCR (qPCR). Simvastatin was found to induce apoptosis successfully in HCC and improve liver fibrosis, overall hepatic function, and necroinflammatory score. Simvastatin, therefore, may be a potential adjunctive therapeutic option in clinical settings of treating HCC.

## Introduction

Hepatocellular carcinoma (HCC) is a primary cause of mortality associated with cancer across the world [1]. The World Health Organization forecasts that more than one million individuals would die from liver cancer by 2030, based on annual figures [2]. In recent decades, significant development has been made in understanding HCC epidemiology, risk factors, and molecular profiles. Furthermore, reasonable methods for prophylaxis, surveillance, early recognition, diagnosis, and therapy have been devised [1]. Until 2006, there were no viable first-line therapy medications for advanced HCC patients, who made up the majority of the total HCC population. Chemotherapy, immunotherapy, hormone drugs, and a variety of other medications were used but gave unsatisfactory results [3, 4]. Chemotherapy for HCC offers certain challenges, such as chemoresistance to existing chemotherapeutic drugs and liver cirrhosis in some HCC patients, which makes treatment with medicines that undergo hepatic metabolism problematic [5]. As a result, research has focused on developing innovative preventive alternatives with significantly higher efficacy to help in the prevention of carcinogenic alterations in the liver and to function as chemosensitizers that increase efficacy and reduce the toxicity encountered with standard anticancer drugs [6, 7].

Apoptosis is a type of programmed cell death, which is tightly controlled at the gene level, culminating in the systematic and effective clearance of dysfunctional cells, such as those that arise after mutations or throughout development [8]. Apoptosis mechanism is complicated, with several signaling channels involved. Cellular apoptosis could be initiated by either the caspase-mediated extrinsic or internal mechanisms. Both routes merge to activate effector apoptotic caspases, resulting in morphological and biochemical intracellular changes, which are signatures of apoptosis [9]. The activation of apoptosis in premalignant lesions as a consequence of mutations might eliminate presumably hazardous cells, hence inhibiting tumor progression. Downregulation of this death mechanism is linked to unregulated cell growth, carcinogenesis, tumor progression, and treatment resistance [10, 11]. As a result, apoptotic dysregulation is regarded as one of the hallmarks of cancer [12]. Therapeutic tactics targeting molecules implicated in apoptotic downregulation are, thus, a viable option to explore in attempt to restore cancer cells’ sensitivity to apoptosis and combat treatment incompetency [13, 14].

Simvastatin is a well-established hydroxy-methylglutaryl coenzyme A (HMG-CoA) reductase inhibitor, a cholesterol-lowering drug, first introduced by Merck and co. in 1988. Simvastatin is a prodrug. It is found in the form of an inactive lactone which is a relatively hydrophobic compound (Manufacturer Information 1991). In the liver, the drug is converted to relatively polar metabolites, which are pharmacologically active [15, 16]. All statins perform their action by blocking HMG-CoA reductase, which is the rate-limiting enzyme in the HMG-CoA reductase pathway, the biological process responsible for endogenous cholesterol synthesis. Statins are more efficacious than other lipid-lowering medications at lowering low-density lipoprotein (LDL) cholesterol, but they are less efficient than fibrates at lowering triglyceride levels. Despite that, statins lower cardiovascular disease incidents and overall mortality regardless of starting cholesterol levels. This is substantial proof that statins act in manners other than cholesterol reduction that is called pleiotropic effects of statins [17].

Several studies highlighted that simvastatin could efficiently induce apoptosis in cancer cells [18–36]. However, the use of simvastatin in HCC treatment was not much spotlighted in literature. Therefore, in this study, the beneficial consequences of simvastatin use in thioacetamide-induced HCC model in rats were addressed. These include the antifibrotic effects, the proliferative inhibitory influence, and the potential to induce apoptosis in HCC cells in vivo.

## Materials and Methods

### Establishment of HCC Model

All animal experiments were carried out following the ethical guidelines of National Institutes of Health (NIH) regarding the care and use of laboratory animals (NIH publication No. 85–23, revised 1985). The experiments were approved by the Research Ethics Committee of Faculty of Pharmacy, Mansoura University, Egypt (approval code #2022–45). Male Sprague–Dawley rats weighing 200–240 g (2–2.5 months of age) were obtained from “Medical Experimental Research Center,” Mansoura, Egypt. Rats were kept for acclimation under standard laboratory conditions of controlled room temperature (25 ± 2 °C) on a 12-h light/dark cycle for 14 days. They were allowed free access to water and food. HCC was established in rats by single 200 mg/kg body weight i.p. injection of diethylnitrosamine (DEN; Sigma Aldrich; St. Louise, MO, USA), followed after 2 weeks by twice weekly i.p. injections of 200 mg/kg body weight thioacetamide (TAA; Sigma Aldrich) for 16 consecutive weeks [37]. The HCC model’s success was verified by liver histopathological evaluation analysis at the completion of the 16-week duration.

### Experimental Design

Rats were divided into three groups as follows: control group (*N*) in which rats were normal and received no treatment (*n* = 10); HCC group in which rats had HCC but received no further treatment (*n* = 12); simvastatin group (SV) which had HCC then received 10 mg/kg simvastatin (granted by EVA Pharma Company for Pharmaceuticals; Cairo, Egypt) by oral gavage daily for 3 weeks (*n* = 12) [38, 39].

### Sample Collection

At the end of the experiment, rats were fasted for 12 h and allowed free access to water. Blood samples were withdrawn from rats through retro-orbital puncture and centrifuged for serum separation. Immediately after sacrificing the rats, the liver was isolated and divided into three sections. The initial section was immersed in RNA-later (Qiagen, Germany) and flash frozen in liquid nitrogen for measurement of gene expression by qPCR. The next liver section was cut in pieces and stored instantly in liquid nitrogen and then in −80 °C refrigerator for later cell cycle analysis. The last liver section was fixed in neutral formalin, embedded in paraffin blocks, and used for histopathological examination and IHC analysis. The liver index was calculated by the following formula: Liver index = [Liver weight (g) / Body weight (g)] × 100 [40]*.*

### Biochemical Analysis

Blood serum collected from animals was used for kinetic colorimetric measurement of alanine aminotransferase (ALT) activity, and also, the level of albumin and total bilirubin by endpoint colorimetric assays.

### Histopathological Examination of Hepatic Tissues

Upon being fixed in 10% neutral buffered formalin, liver tissue sections were integrated in paraffin blocks and cut into 4-μm-thick sections for histological analysis under a light microscope with hematoxylin and eosin (H&E) and Masson’s trichrome stains. H&E-stained hepatic sections were utilized to assess necroinflammatory scores, which were guided by the Ishak modified histology activity index [41]. Necroinflammatory changes were calculated in H&E-stained liver sections as the sum of four categories: periportal or periseptal interface hepatitis (0–3); necrosis (0–3); ballooning degeneration (0–4); steatosis (0–3); and fibrosis (0–3).

Liver sections were also stained with Masson’s trichome stain for semi-quantification of collagen fiber deposition in liver tissues [42], using the ImageJ software and the fibrosis percentage was used to assess the fibrosis degree. The percentages for central vein thickening, the portal area expansion, and parenchymal fibrous areas from the ten fields each were expressed as the mean ± standard error. The total fibrotic area for each sample was calculated as the mean percentage of the three zones.

### Immunohistochemical Analysis

Four-micrometer-thick liver sections were deparaffinized with xylene and then rehydrated in descending ethanol concentration (100–95-75%). Epitope retrieval was carried out by heat-induced epitope retrieval (HIER) technique using the Cell Marque triology™ with pressure cooker. After rinsing with 5 changes of distilled water and PBS, antigen retrieval was enhanced by heating in citrate buffer pH 6. For blocking endogenous peroxidase activity, 3% H_2_O_2_ was used. The tissue thereafter was incubated with the corresponding antibodies for 1 h. For immunohistochemical analysis of caspase-3, primary antibodies against active caspase-3 (GB11532) (Servicebio, China dilution 1:1000 catalog # GB11532) were used. And further incubated with Ultra Vision HRP polymer for 15 min. A previously prepared 3,3′-diaminobenzidine (DAB) substrate solution was used to cover each slide. The solution was made up of a 1:1 mixture of DAB chromogen solution and DAB buffer solution. Washing by distilled water, counter-staining using hematoxylin, and dehydration by xylene and escalating degrees of ethanol were the final three stages. Eventually, the slides were examined under a light microscope. The ImageJ software was used to analyze the images and compute stain intensity using the area fraction approach.

### Calculation of Apoptotic Index

Counts of apoptotic cells and apoptotic bodies were performed using a light microscope total magnification of 400× (high power), a 40× objective lens, and a calibrated eye-piece lens (field diameter 0.4 mm). Liver cells in 10 fields distant from necrotic zones were examined. Then, the number of labeled cells in immunostained sections were counted. The apoptotic index was defined as the total number of apoptotic cells per 10 high power fields (HPFs) [43].

### Cell Cycle Analysis by Flow Cytometry

Fresh liver tissue specimens were transported in isotonic saline pressed through a nylon grid then treated with isotonic Tris-EDTA buffer containing RNAse (1 mg/mL). The cell suspension was centrifuged and the supernatant was aspirated. Suspensions of single cell nuclei were obtained by pepsin; these procedures were according to Tribukait [44]. The fluorochrome stain was prepared by adding 25 μg/mL of propidium iodide (PI), 500 mg sodium citrate, and 0.5 ml Triton X-100 to 500 mL distilled water. One milliliter of cell suspension was suspended in 1 mL of the staining solution at 4°C, for 30 min, and then filtrated through 30-μm nylon mesh to eliminate nuclear clumps immediately before analysis [45, 46]. The staining cell was kept in dark for at least 24 h for determination of the subG_1_ phase; then, the samples were analyzed at accuri™ C6 BD flow cytometer (Biosciences, San Jose, CA, USA) by using the accuri™ C6 software for analysis and acquisition of data.

### Quantitative Real-Time Polymerase Chain Reaction (qRT-PCR)

Liver tissue (50 mg) was processed in accordance with the manufacturer’s instructions of GeneJET RNA purification kit (Thermo Fisher Scientific, USA) to extract total RNA for subsequent gene expression analysis of Bcl2, caspase-3, and caspase-8. Genomic DNA was eliminated from the RNA samples by Dnase 1, Rnase-free kit (Thermo Fisher Scientific, USA). Extracted RNA was measured and purity was verified using Nanodrop 2000 UV–Vis spectrophotometer (Thermo Fisher Scientific, USA). One microgram of total RNA was reverse transcribed to its complementary DNA (cDNA) using high capacity cDNA reverse transcription kit (Thermo Fisher Scientific, USA) by utilizing PCR Thermal Cycler TCA0096 (Thermo Fisher Scientific, USA).

This was accomplished with the use of a PCR thermal cycler. PCR primers were designed using the Refseq-RNA PubMed primer blast, Primer3, and NetPrimer websites in line with the gene sequence from PubMed (Entrez Gene) and blasted to guarantee specificity with the target gene using the NCBI/BLAST website. The primer sequences are listed in Table 1. RT-PCR reactions were performed on a Real-time PCR system StepOne PlusTM, applied biosystems (Thermo Fisher Scientific, USA) using the SensiFastTM SYBR®-No ROX kit (Bioline, USA) following the instructions manual. Rat glyceraldehyde 3-phosphate dehydrogenase (GAPDH) was employed as a housekeeping gene. Ct data for gene specimens were standardized against GAPDH as an internal reference, and relative gene expression of the genes that were investigated was computed using the 2^−ΔΔCt^ method.

**Table 1:** Sequences of qPCR primers

Gene of interest		Primer sequence	Accession number	Product size	Reference from literature if available
Caspase-3	Forward	5′-GGAGCAGTTTTGTGTGTGTGA-3′	NM_012922.2	191	[47, 48]
	Reverse	5′-TGTCTCAATACCGCAGTCCA-3′			
Caspase-8	Forward	5′-CCTTTCTCCTCCCTCTGACCTC-3′	NM_022277.1	193	[47]
	Reverse	5′-GTAACCTGTCGCCGAGTCCC-3′			
Bcl-2	Forward	5′-AGGATAACGGAGGCTGGGATG-3′	NM_016993.1	179	[47]
	Reverse	5′-TATTTGTTTGGGGCAGGTCT-3′			
GAPDH	Forward	5′ TCCCATTCTTCCACCTTTGA -3′	NM_017008.4	109	-
	Reverse	5′ – CCACCACCCTGTTGCTGTAG – 3′			

### Assessment of Serum Alpha-Feto Protein (AFP) by ELISA

Serum AFP was measured according to manufacturer’s instructions using AFP ELISA Kit (precheck, USA, Lot AFP37312C) and expressed as ng/ml.

### Statistical Analysis

All results were expressed as mean ± S.E.M. Experimental results were examined using one-way analysis of variance (ANOVA) followed by Tukey’s post hoc test. Necroinflammatory and immunohistochemical scoring results were analyzed using the non-parametric Kruskal–Wallis test followed by Mann–Whitney post hoc test. Statistical tests were performed by using the GraphPad Prism 2019 v8.0.2.263 software (IBM Corp., Armonk, NY, USA). Statistical significance was considered at *p* < 0.05.

## Results

### Simvastatin Improved Hepatic Architecture and Liver Function Biochemical Tests in HCC

**Fig. 1:**
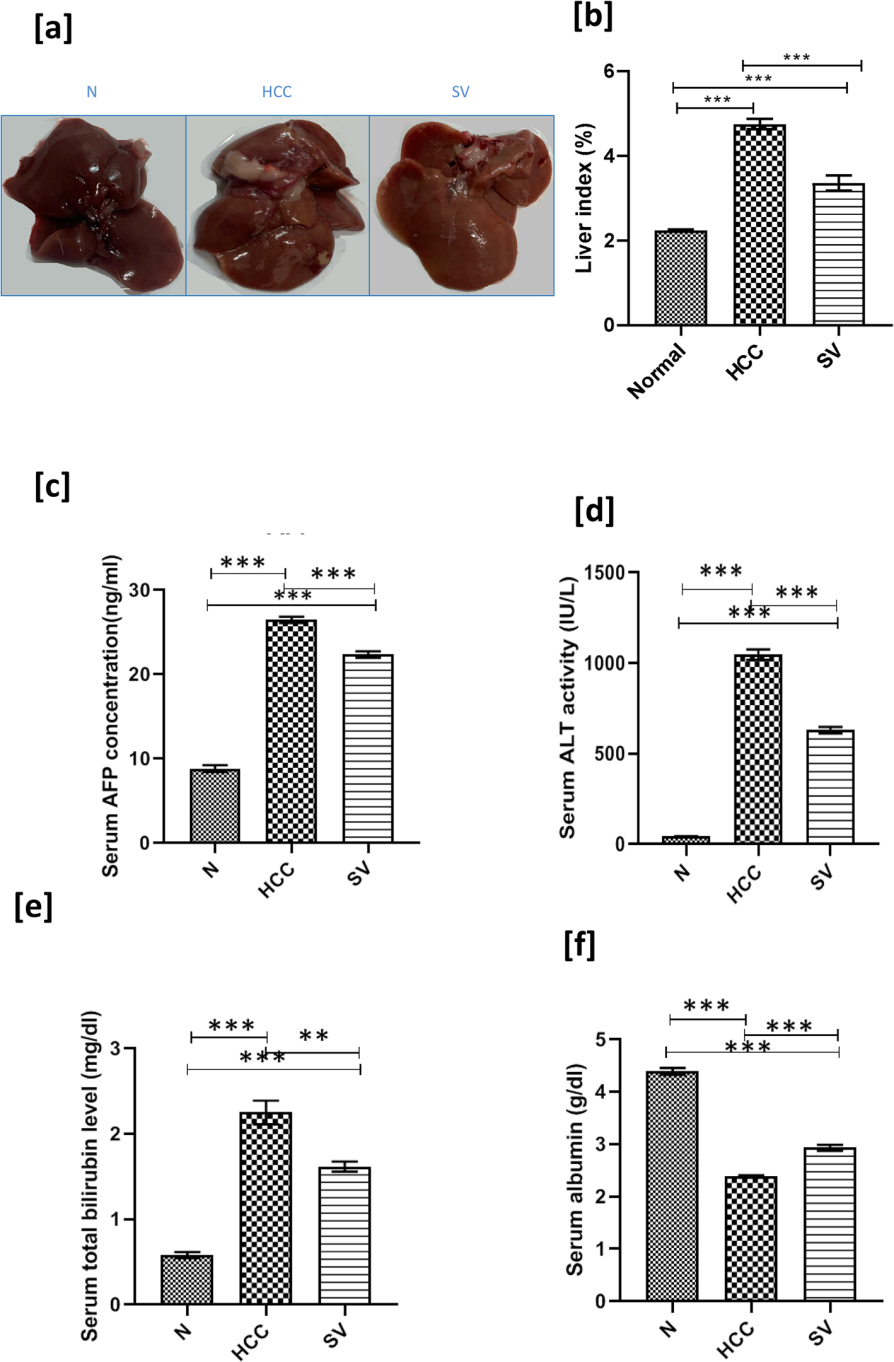
**a** Liver gross examination images of livers from control (*N*), hepatocellular carcinoma (HCC), and HCC treated with simvastatin (SV) groups. **b** Liver index at the end of the study in different groups (liver to body weight%). **c** Elisa measurement of serum AFP expressed in ng/ml. **d** Serum activity of alanine aminotransferase (ALT). **e **Serum level of  bilirubin.** f **Serum level of albumin, at the end of the study in different groups; *p˂0.05; **p˂0.01; ***p˂0.001

Gross examination of liver from the HCC group showed relatively rough nodular surface with noticeably faint discoloration when compared to smooth reddish appearance of liver from the *N* group. SV showed smoother and less discolored liver relatively to the HCC group (Fig. 1a).

Simvastatin reduced liver index when compared to the HCC group (*p* < 0.001) (Fig. 1b). Not only serum ALT was reduced by simvastatin by 0.6-fold when compared to the HCC group (p < 0.001) (Fig. 1d), but also lowered that of bilirubin, by 0.7-fold (*p *< 0.01) than that of HCC (Fig. 1e). Additionally, serum albumin increased by 1.23-fold compared to that of HCC (*p* < 0.001) (Fig. 1f).

In regard to HCC standard tumor marker AFP, simvastatin reduced serum AFP by 0.85-fold when compared to the HCC group (p < 0.001) (Fig. 1c).

### Simvastatin Improved Necroinflammatory Score, Histopathological Examination, and Fibrosis Degree in HCC

**Fig. 2:**
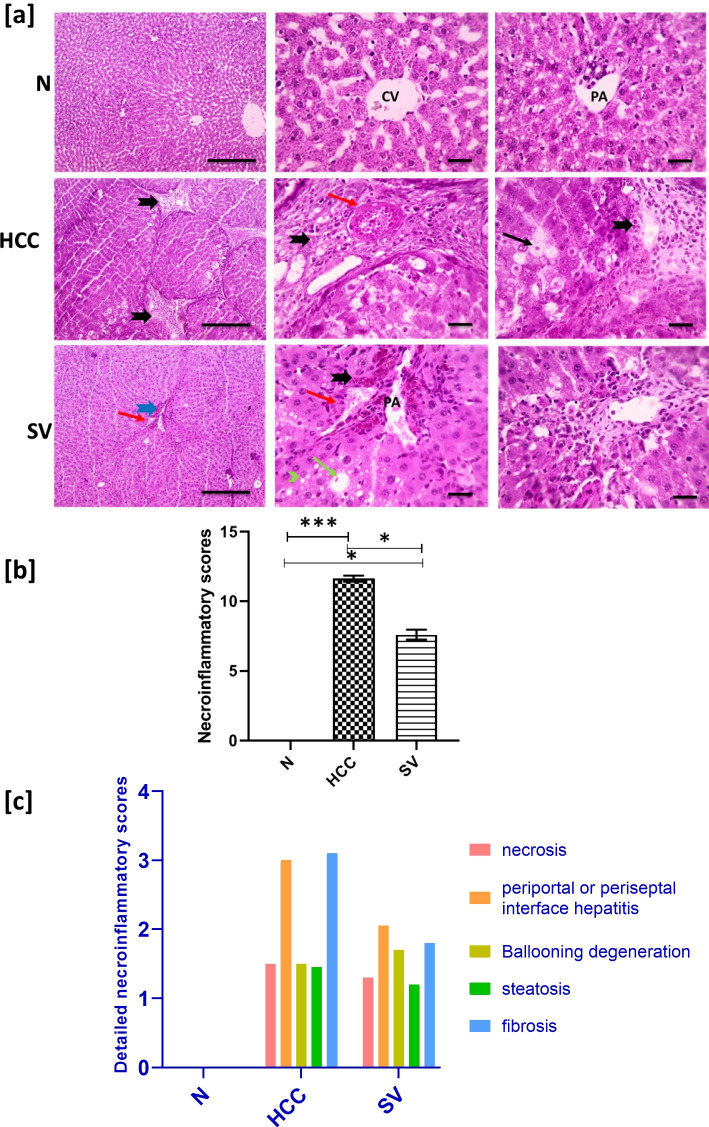
**a** Representative microscopic images of H&E-stained hepatic sections from control (*N*), hepatocellular carcinoma (HCC), and HCC treated with simvastatin (SV) groups, central veins (CV), portal areas (PA), leukocytic cells and hemosiderin laden macrophages (thick black arrows) ballooning degeneration in hepatocytes (thin arrows), fibrous strands (thick blue arrows), congested blood vessels (red arrows), macro-vesicular steatosis (green arrow), micro-vesicular steatosis (green arrowheads), and low magnification ×100 bar and high magnification ×400 bar. **b** Statistical representation of necroinflammatory score. **c** Detailed necroinflammatory score. *p˂0.05; **p˂0.01; ***p˂0.001

Microscopic pictures of H&E-stained liver sections showed normal arrangement of hepatic cords, central veins, portal areas, and sinusoids in the control group (*N*). Liver sections from the group (HCC) showed marked disruption of arrangement of hepatic cords, central veins, and portal areas characterized by marked thick anastomosing fibrous tissue deposition containing congested blood vessels, leukocytic cells, and hemosiderin laden macrophages, forming multiple separate complete hepatic nodules with ballooning degeneration in hepatocytes. The liver section from the treated group (SV) showed markedly decreased hepatic lesions characterized by very thin short fibrous strands extending from portal areas, containing few congested blood vessels and hemosiderin laden macrophages. Few macro- and many micro-vesicular steatosis are seen in hepatocytes (Fig. 2a).

Also, the histopathological examination of H&E-stained liver sections revealed lower necroinflammatory score in SV by 0.6-fold (*p *< 0.05) when compared to HCC (Fig. 2b). This score is a sum of four categories, where the strongest decrease was in the fibrosis and hepatitis degrees when compared to HCC (Fig. 2c).

**Fig. 3:**
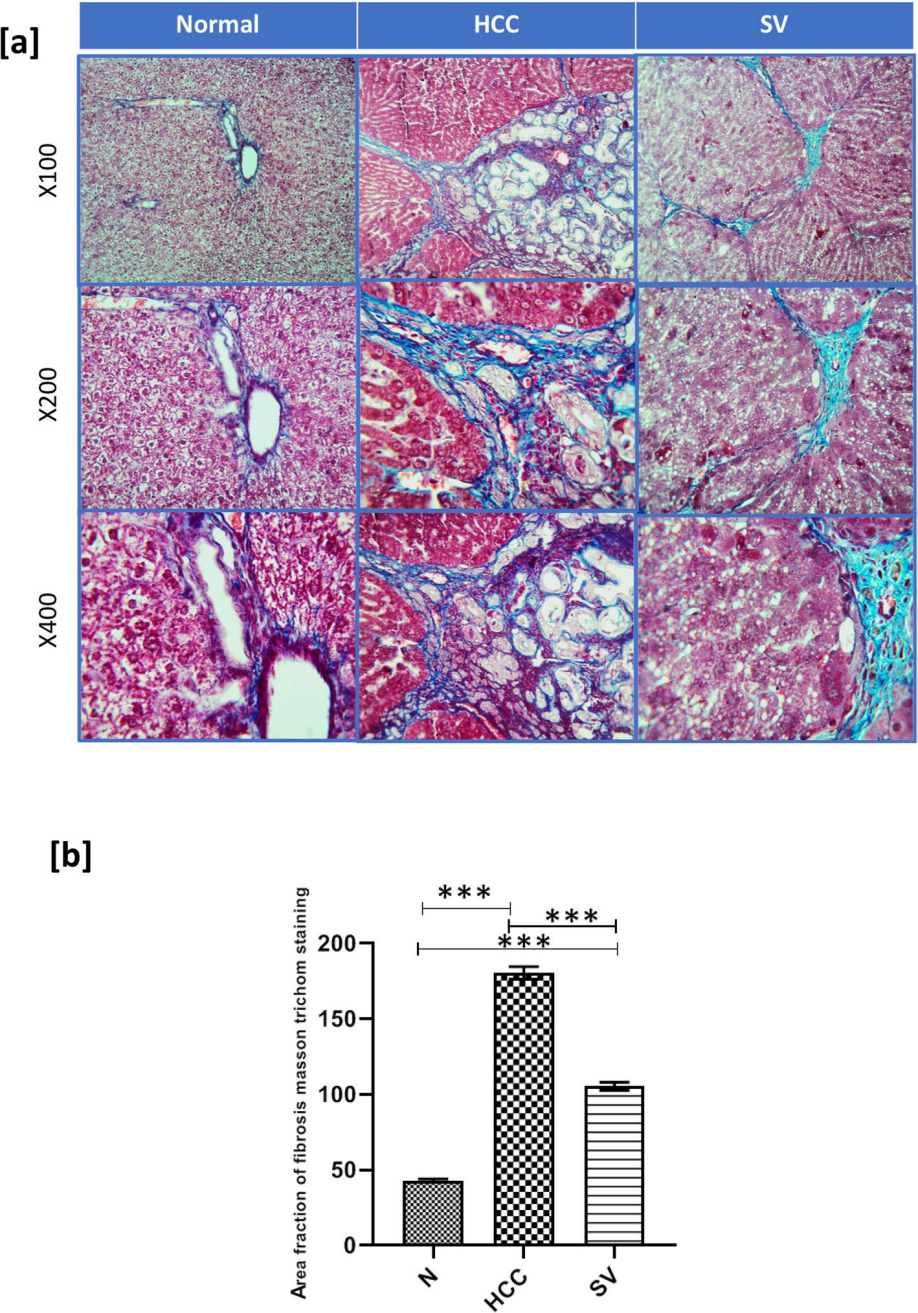
**a** Representative microscopic images of Masson trichome-stained liver sections from control (*N*), hepatocellular carcinoma (HCC), and HCC treated with simvastatin (SV) groups, upper panel ×100, and lower panel ×400. **b** Mean fibrosis area fraction using Masson trichome staining; *p˂0.05; **p˂0.01; ***p˂0.001

Masson’s trichrome-stained liver sections manifested less collagen distribution (Fig. 3a) and milder fibrosis area fraction in the SV group when compared to HCC group (*p* < 0.001) (Fig. 3b).

### Simvastatin Induced Apoptosis in HCC

**Fig. 4:**
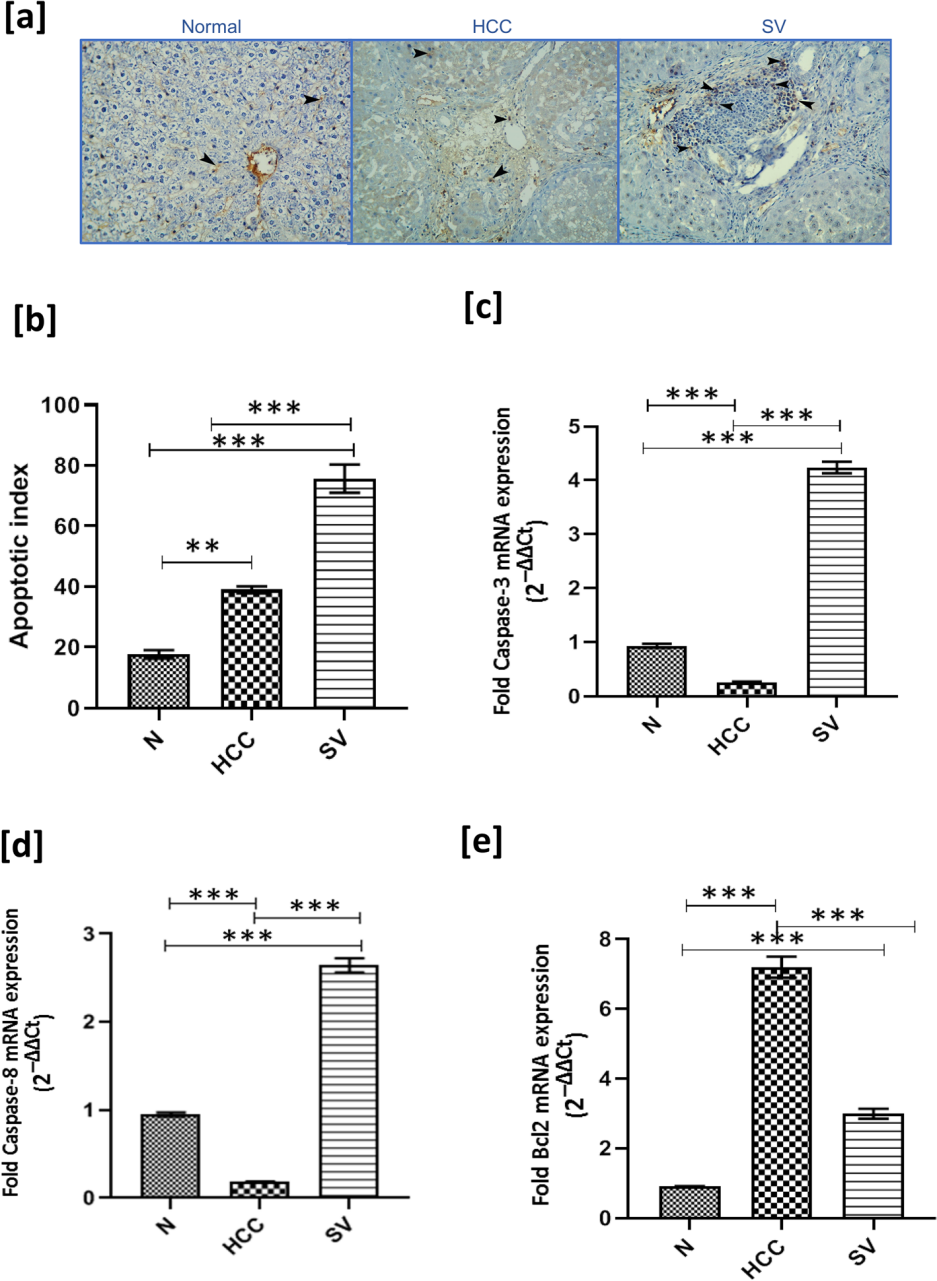
**a** Representative figures of active caspase-3 protein (brown color) at the end of the study from control (*N*), hepatocellular carcinoma (HCC), and HCC treated with simvastatin (SV) groups, counterstained with hematoxylin; apoptotic cells (black arrow heads) upper panel: × 100 bar; lower panel: × 400 bar. **b** Statistical analysis of caspase-3 apoptotic index at the end of the study in different groups. **c–e** Relative mRNA expression (2^−ΔΔct^) of **c** caspase-3, **d **caspase-8, and **e** Bcl-2 normalized to GAPDH at the end of the study in different groups; *p˂0.05; **p˂0.01; ***p˂0.001

- IHC staining of active caspase-3 showed very mild staining in the *N *group, and slightly stronger but insignificant staining in HCC (*p* = 0.3); on the other hand, the SV group showed significant increase in brown staining (*p* < 0.001) when compared to HCC (Fig. 4 a and b).
- On the transcriptional level of apoptotic proteins, SV treatment resulted in upregulation of caspase-3 and caspase-8 gene expression, while it downregulated the anti-apoptotic protein Bcl2 (*p* < 0.001) (Fig. 4 c, d, and e).

### Simvastatin Caused G_0_/G_1_ Cell Cycle Arrest and Switched Cell Division into Cell Death

The simvastatin treated group showed accumulation of cells in subG_1_ (*p* < 0.001). Apoptotic or necrotic cells are represented by the subG_1_ phase (fragmented DNA that appears on the graph before the G_1_ phase).

**Fig. 5:**
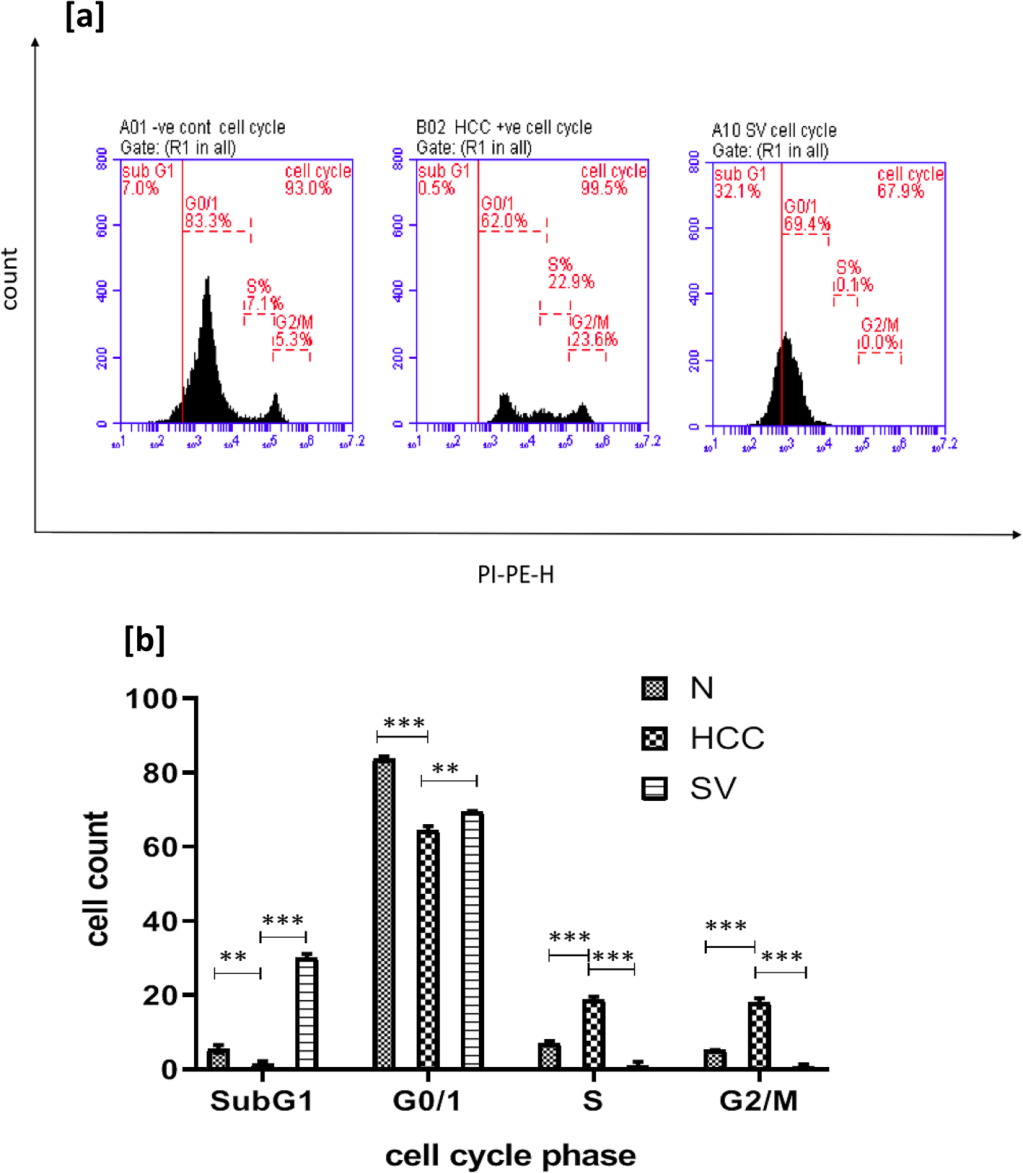
**a** Statistical representation of cell cycle analysis results at the end of the study in control (*N*), hepatocellular carcinoma (HCC), and HCC treated with sorafenib (SF) or simvastatin (SV) groups. **b** Representative figure from cell cycle analysis charts at the end of the study in different groups; *p˂0.05; **p˂0.01; ***p˂0.001

Additionally, we noted that simvastatin caused G_0/1_ cell cycle arrest which is seen as the accumulation of more percentage of cells in the resting state (*p* < 0.01) rather than replication phases when compared to the HCC group. On contrary, the HCC untreated group had minimal cells in the subG_1_ phase and high percentage in S and M phases indicating high degree of mitosis, the thing that was very diminished in the SV group (Fig. 5a and b).

## Discussion

HCC is among the greatest rising causes of cancer-related death worldwide, and the prevalence of HCC is tragically on the rise [49]. Because of the increasing incidence and poor outcome of HCC, prophylaxis is a plausible approach to lowering death rates linked with this malignancy [50]. Chemotherapy for HCC offers certain challenges, such as chemoresistance to existing chemotherapeutic drugs and liver cirrhosis in some HCC patients, which makes treatment with medicines that undergo hepatic metabolism problematic [5]. As a result, research has focused on developing innovative preventive alternatives with significantly higher efficacy to help in the prevention of carcinogenic alterations in the liver and to function as chemosensitizers that increase efficacy and reduce the toxicity encountered with standard anticancer drugs [6, 7].

Thioacetamide (TAA) has been recognized as a strong hepatotoxin since 1948, causing centrilobular necrosis and an upsurge in circulating transaminases and bilirubin [51]. Prolonged exposure causes cirrhosis and HCC in experimental animal models [52]. TAA is transformed to TAA-sulfoxide, which is then metabolized to produce a far more reactive S,S-dioxide derivative capable of attacking lipids and proteins. TAA oxidation produces reactive metabolites and free radicals, which are linked in changing cell permeability and limiting mitochondrial action, resulting in cell death [51, 53, 54].

Accordingly, we used TAA 200 mg/kg body weight i.p. twice per week for 16 consecutive weeks after 2 weeks of DEN single-dose injection, to achieve the closest pathway possible that occurs in the majority of HCC patients, fibrosis, then cirrhosis, and eventually carcinogenesis [37].

In the present study, the serum ALT activity was significantly elevated in the HCC group as compared to the control group. The increased activity of ALT enzyme was a consequence of hepatocyte membrane damage causing alterations in both cellular and mitochondrial membrane permeability [55]. This is in agreement with [47, 48, 56–59] who proved that ALT activity was significantly increased in HCC induced by thioacetamide in rats as compared to normal control rats. Simvastatin treatment reduced the degree of hepatocellular damage evidenced by decreased serum ALT level.

Hepatic damage is also associated with elevated bilirubin concentration as well as decreased total protein and albumin concentration [60, 61]. In our study, serum albumin was decreased in addition to the elevation of serum bilirubin. SV managed to improve hepatic synthetic function as well as detoxification function evidenced by increasing serum albumin and decreasing serum bilirubin.

AFP is an oncofetal serum glycoprotein present at birth and eventually absent in all healthy adults. However, AFP is generally overexpressed during hepatic inflammation and precancerous stage as well as in hepatic cancer cells and is usually employed as a diagnostic marker for HCC [62–65]. The significant high levels of AFP are usually related to poor differentiation and biologically malignant characteristics especially portal vein invasion of HCC [66, 67]. In the current study, serum AFP concentration was significantly elevated in the HCC group as compared to the control group, in agreement with [47, 48, 57, 60] who reported that AFP concentration was significantly increased in TAA-induced HCC rats. The SV group exhibited lower serum AFP levels (*p* < 0.0001), increasing the probability of better prognosis of HCC.

In the present study, histopathological examination of liver tissues revealed a significant increase in fibrosis percentage and collagen deposition in the HCC group compared to the control group as indicated in Masson’s trichrome-stained liver sections. Moreover, as indicated in hematoxylin and eosin (H&E)-stained liver sections, marked necroinflammatory changes, as well as several large dysplastic and neoplastic tumor nodules, have been reported in the liver of rats receiving TAA. The SV group showed better histopathological score and less collagen deposition and fibrosis degree.

For decades, a hallmark and goal of clinical oncology have been the development of drugs and protocols that assist the effective destruction of cancerous cells by apoptosis. In regard to apoptotic index, the HCC group showed mild insignificant apoptosis induction, which can be explained by the fact that apoptosis is a programmed cell death activated upon DNA alteration or mutation as in the case of carcinogenesis early stages, while on the level of gene transcription of caspase-3, caspase-8, and Bcl-2, we found very reduced relative expression to the normal group in the case of caspases and elevated relative expression to normal in the case of Bcl-2, probably by negative feedback inhibition of caspases and positive feedback stimulation of Bcl-2 by cancer cells in later stages of cancer progression.

When compared to the untreated HCC group, the SV treatment group showed a significant increase in all of the apoptotic signs, early and late in the pathway, indicating increased cytotoxicity, and hence its efficacy as an anticancer drug. These results are in agreement with the numerous studies which indicate that simvastatin could induce apoptosis in different types of cancer cells [18–36].

In the cell cycle analysis, the simvastatin treatment caused the proportion of cells in S and G_2/M_ phases to decrease with a high peak in the subG_1_ phase. This was suggesting that the lost percent from S and G_2/M_ phases was replaced by apoptosis and to lesser extent towards the resting state. This means that instead of the uncontrollable state of replication in form of mitosis seen in the HCC group, simvastatin managed to induce apoptosis to cancer cells and caused G_0/1_ cell cycle arrest.

Although the studies done on experimental animals in vivo provide a close representation to the complexity of living organisms rather than studies done on isolated cells, one limitation is the inability to assess the pharmacological effects on the cancer cells alone as accurate as in cell line studies. In this study, the specimen used were liver sections which include both normal hepatocytes and carcinogenic ones. On top of that, animals show high individual variability between them.

In a nutshell, simvastatin showed ameliorative influences on HCC in regard to tumor mass, serum AFP, liver function tests, hepatitis, fibrosis, dimensioned apoptosis, and unrestrained cancer cell proliferation.

## Conclusion

**Fig. 6:**
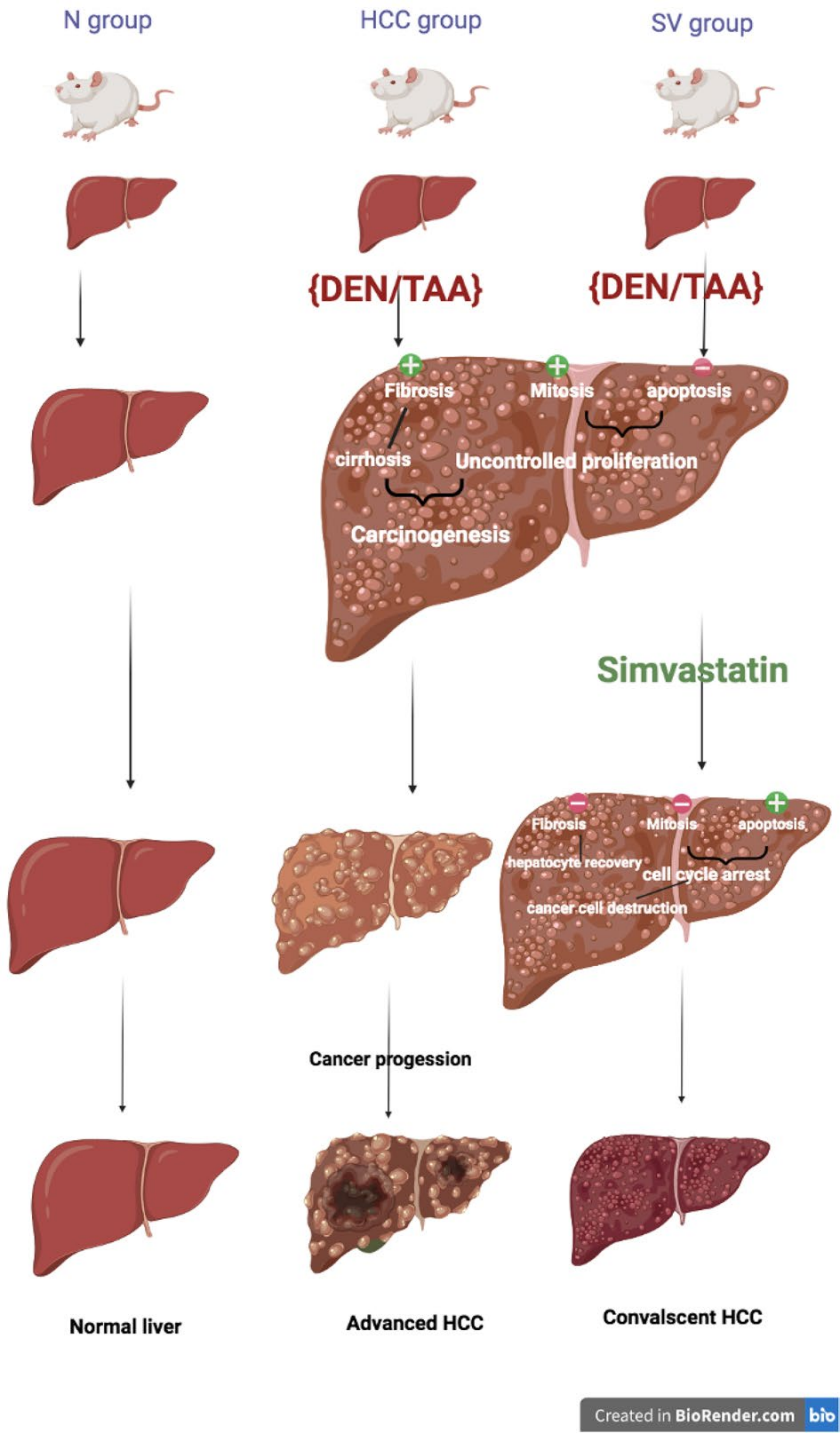
Proposed mechanism of simvastatin (SV) in hepatocellular carcinoma (HCC) induced by diethylnitrosamine (DEN) and thioacetamide (TAA) in comparison to the HCC group and normal control (*N*)

In aggregate, the simvastatin treatment of HCC decreased the tumor mass by inhibiting cancer cell proliferation, inducing apoptosis, and allowing the liver to enter the convalescent state by its antifibrotic effect (Fig. 6). Therefore, we recommend that simvastatin, the safe antihyperlipidemic drug, should be considered an adjuvant drug in HCC treatment protocols.

## Data Availability

Not applicable

## Abbreviations

HCC: Hepatocellular carcinoma
DEN: Diethylnitrose amine
TAA: Thioacetamide
AFP: Alpha-feto protein
IHC: Immunohistochemistry
qPCR: Quantitative PCR
